# Hit or Miss: Fertilization Outcomes of Natural Inseminations by Japanese Quail

**DOI:** 10.1371/journal.pone.0131786

**Published:** 2015-07-29

**Authors:** Elizabeth Adkins-Regan

**Affiliations:** Department of Psychology and Department of Neurobiology and Behavior, Cornell University, Ithaca, New York, United States of America; Utrecht University, NETHERLANDS

## Abstract

Variation in fertilization success underlies sexual selection, yet mating does not guarantee fertilization. The relationship between natural inseminations and fertilization success is essential for understanding sexual selection, yet that relationship and its underlying mechanisms are poorly understood in sperm-storing vertebrates such as birds. Here the relationship is analyzed in mating trials using Japanese quail (*Coturnix japonica*), which show striking variation in the fertilizing success of inseminations. Failures of males’ inseminations to fertilize eggs were mainly due to failures prior to sperm-egg contact. Fertilization probabilities on any given day were unrelated to whether the female had laid an egg the previous day, arguing against stimulation of sperm release from sperm storage tubules by the events of the daily egg-laying cycle. Instead, an unfertilized egg laid between two fertilized eggs predicted a longer sperm storage interval. Both sexes gained similar numbers of fertilized eggs by mating with a second partner the next day, but males, unlike females in a previous study, did not gain by having two females to mate with at the same time. Instead, they were both behaviorally and sperm limited, whereas females gain by mating twice in quick succession. Even double inseminations often failed to fertilize any eggs, and multiple matings would be needed for an entire clutch to be fertilized with high certainty. Paradoxically, this low and probabilistic fertilization success co-occurs with other notable characteristics of male quail suggestive of past sexual selection for increased success, including vigorous copulatory behavior, forced copulations, foamy secretion aiding in sperm competition, large testes and unusual sperm morphology.

## Introduction

Sexual selection occurs through variation in mating and especially fertilization success [1, 2]. Understanding the mechanisms underlying that variation is therefore central to further advances in the field. It has long been known that in many animals such as birds a male’s fertilization success (as indicated, for example, by paternity) cannot be judged from observations of mating success [3, 4], so that mating and fertilization success are two different components of reproductive success. To succeed, the male not only has to mate (the stage for pre-copulatory sexual selection) but also to ejaculate and inseminate the female (transfer sperm and accessory fluids to her reproductive tract, assuming internal fertilization). Then those sperm must encounter and penetrate an ovum, the two genomes must combine appropriately, and embryonic development must proceed normally [5]. There is some variable probability of failure at each step in this series even when there is no sperm competition (post-copulatory sexual selection). Among mammals, for example, a single mating sometimes produces a pregnancy in rodents (see, for example, a study with *Microtus californicus* [6]) but seldom does in humans [7], a species difference due in part to the fact that female rodents only mate close in time to ovulation. Then in male-male competitive situations, failure probabilities may be increased, and post-copulatory (fertilization) success is not necessarily predicted by pre-copulatory (mating) success (e. g., [8]).

The relationship between the insemination and fertilization steps in the series is particularly interesting to consider in the many vertebrates such as birds in which females can store sperm [9]. Whether the male will fertilize eggs will depend, along with other factors, on whether his sperm were stored, how long they have been stored, and whether the stored sperm were released in sufficient numbers at the right time for fertilization [10]. Sperm storage also provides a potential way for the female tract to bias whether the sperm are used or even which male’s sperm are used following multiple mating, that is, to exercise post-copulatory (cryptic) female choice between male ejaculates. Although good evidence for this particular phenomenon is thus far largely limited to invertebrates, nonetheless it is more generally the case that when fertilization is internal, the female reproductive tract is the context in which sperm competition and fertilization occur [11].

In most birds with clutch sizes of two or more, one egg is laid each day until the clutch is complete [5]. The ovulation for the next egg occurs shortly after oviposition, and the ovum is fertilized in the upper oviduct. Sperm are stored in sperm storage tubules (SSTs) located mainly at the utero-vaginal junction, with possible additional storage at the infundibulum in the upper oviduct, the site of fertilization. Sperm are released from the SSTs to swim up to the fertilization site, and can still fertilize eggs after storage in SSTs ranging from a few days to several months, depending on the species [5, 12].

How sperm transfer to the female during mating (natural insemination) translates into fertilization success in birds is poorly understood. Wild species are not well suited to such research because of the difficulty of accurately knowing whether and when mating occurred and whether it resulted in insemination. Hatching rates in wild clutches can be well below 100%, and whether this is due to failure of stored sperm to fertilize eggs is seldom known [13]. Even in domesticated birds such as chickens that have been subject to artificial selection for high reproductive efficiency, many matings do not fertilize any eggs [14].

In order to better understand the relationship between natural insemination and fertilization outcome in a sperm-storing bird and its mechanistic basis, a series of experiments was carried out using Japanese quail. Both domestic and genetically wildtype birds have low fertilization success from matings (see below and methods section). Females of this species ovulate and oviposit on a daily basis, and can store sperm for up to 11 days [15, 16]. A major advantage of this species is the ease with which matings can be arranged for experimental purposes. Male Japanese quail that are housed singly are highly sexually motivated and once habituated to the mating cage most will initiate mating with a female within a few seconds, seldom requiring more than five minutes [17]. Another advantage is that insemination can be determined quickly and non-invasively. Male quail produce large quantities of white foam from the foam gland (also called cloacal or proctodeal gland, located dorsal to the cloacal opening) that is transferred to the female along with the semen. The male’s foam both enhances his fertilization success in the absence of competition and also provides him with a sperm competition advantage in competitive mating situations [18, 19]. Foam is very seldom present in the female unless semen also is [17, 20]. Thus insemination can be confirmed by the sight of the white foam in the female’s cloaca or in the drop pan under her home cage during the period following mating. Even when insemination is confirmed, however, fertilization outcomes are still highly variable, with over one-third of inseminations fertilizing no eggs at all (complete fertilization failure) and the rest fertilizing a variable number ranging between 1 and 10 eggs [21]. Paradoxically, this low and variable success co-occurs with other characteristics, including the unique foam, that would be expected to favor fertilization. Male quail have quite large testes for their body size compared to other birds [22–24], produce spermatozoa much more quickly than mammals [25] and produce sperm with an unusually large midpiece (the energy producing mitochondria containing portion) compared to other galliforms [26]. Because outcomes of repeated trials with the same male and different females, or the same female and different males, are uncorrelated, variability does not seem to be caused by some males being less fertile or some females being harder to fertilize [21]. In another galliform bird with a different social system, *Gallus gallus*, females’ ejections of inseminations reduce male fertilization success, but this does not seem to be the case in Japanese quail [21, 27]. The clutch size in wild populations is 5–13 [28]. Thus multiple successful inseminations would be required to guarantee fertilization of an entire clutch, providing ample opportunity for both sexes to increase their reproductive success through multiple mating.

The experiments reported here were designed to address the following questions about the variable relationship between natural insemination and fertilization in Japanese quail:

1. Does the large number of complete fertilization failures occur because sperm failed to reach and penetrate the ovum, or because processes following sperm penetration failed? (Experiment 1)
2. How do fertilization probabilities change across the daily egg laying sequence, and how are those probabilities affected by a skipped egg in the sequence? Birkhead and Fletcher [16] determined the decline in fertilization probability following removal of female quail from mixed-sex housing, where the number and recency of matings was unknown. Here the probability trajectory following single matings with confirmed inseminations was determined. In addition, fertilization probabilities were determined following a day when no egg was laid. Such laying gaps occur occasionally, for unknown reasons, even in female quail with high egg production, and are a characteristic feature of the laying sequences of a number of wild bird species [29]. They provide an opportunity to test the hypothesis that events of the daily egg cycle (ovulation, oviposition or associated hormonal pulses) might trigger sperm release, as was proposed for chickens and quail [30–33]. (Experiment 2)
3. Do quail gain reproductive success (achieve more fertilized eggs) with a second insemination? How do males and females compare with respect to any gain? Does it make a difference whether the female mates with two different males rather than the same male twice? (Experiments 3 and 4)
4. Can males gain by having two females to mate with simultaneously, or do they gain only if the females are spaced apart in time? Observations of the sibling species (*Coturnix coturnix*) and of genetically wildtype Japanese quail suggest flexible mating systems consisting of short-term pair relationships with mate switching and extra-pair matings [34, 35]. In favorable habitats both *Coturnix* species occur at high enough densities for males to encounter multiple females close together in time [28]. Can they actually fertilize two females in quick succession and increase their reproductive success that way? Or are they sperm limited? Domestic male *Coturnix japonica* have high sperm production rates [25], and fertilization success is not reduced by mating the day before (shown in Experiment 4). Whether they are sperm limited when mating twice close together in time is not known, however. (Experiment 5)

## Materials and Methods

### Animals

Quail were hatched from fertilized eggs obtained from CBT Farms, Chestertown, MD. Eggs were supplied so as to avoid full siblings. Beginning at age 4 weeks, all birds were housed individually on a 16L:8D light:dark cycle to initiate and maintain reproductive activity. Subjects in the experiments were at least 8 weeks old, sexually mature (developed foam glands in males, regular egg laying by females), sexually experienced (had mated one or more times) and less than one year old. Males and females were housed so they could not see each other. Each experiment used different birds from different hatching cohorts, and no birds had been used in any prior experiment.

### Ethics statement

All animal housing, use, procedures and studies reported here were approved by the Institutional Animal Care and Use Committee of Cornell University (protocol numbers 2002–0117 and 2012–0098).

### Assessment of insemination and fertilization success

All mating trials were carried out between late morning and mid-afternoon. Males were allowed to mate once with the assigned female and whenever the mating appeared to be complete judging from his behavior (cloacal contact followed immediately by cessation of the mating attempt), insemination was confirmed by the presence of white foam in the female’s cloaca or drop pan afterward, as described above.

Eggs are laid in the late afternoon or early evening, and there are nearly 24 hours between the time a particular ovum is fertilized and the time that egg is laid. The first egg that could possibly be fertilized by a mating is the egg laid the day after mating, which, if laid late in the day, would be found the next morning, on day 2 after mating on day 0. Therefore, eggs were collected daily in the mornings of days 2 through 11. Eggs were stored at 7.2°C prior to incubation at 37.5°C and approximately 30% relative humidity. After one week of incubation, they were broken open to check for the presence of an embryo. Early embryonic deaths were uncommon (1 to 3% of eggs) and unless stated otherwise were counted as fertilized eggs. Trials of females that did not go on to produce at least five intact (unbroken) normal (not soft-shelled) eggs were discarded.

The quail in the experiments are domesticated birds. It is unlikely that fertilization failures are due to domestication, however, because artificial selection for food production normally results in increased, not decreased, reproductive efficiency. Nonetheless, before beginning the experiments it was important to find out whether the low and variable fertilization success is also typical of less domesticated quail. Therefore, a pilot study was carried out using a line of Japanese quail obtained from the University of British Columbia (UBC) that is descended from wild caught stock (http://www.landfood.ubc.ca/avian_research/avian_genetic_pgm.htm). This line originated from populations introduced into the Hawaiian Islands in 1921 and 1944. They had been free-living there for 40–60 years. The sub-population from which the UBC Hawaiian line birds were descended was trapped from the island of Hawaii in 1985 and maintained at the UBC Quail Genetic Resource Centre (now the Avian Genetic Resource Laboratory). One generation per year had been produced by letting birds breed in outdoor flight pens, with no other human management of breeding [34]. For the pilot study, fertilized eggs of the UBC Hawaiian line were obtained and birds were hatched and reared in the laboratory. After sexual maturity was reached, five male Hawaiian line quail were mated with eight female Hawaiian line quail for a total of 10 mating trials. Trials took place in a wire mesh cage 1.8 m X 0.8 m X 0.9 m (height). Males were allowed one insemination per trial, and eggs were collected and incubated as described above. Males copulated vigorously, and there were forced copulations, as with domestic males, yet fertilization success was low. Only three of the 10 trials resulted in any fertilized eggs, and only one egg was fertilized in each of those three trials. Thus, as expected, fertilization success was lower, not higher, than in prior work with domesticated birds [21]. The low fertilization success of single matings and single inseminations appears to be a species characteristic, not a product of domestication.

### Statistics

Fertilization outcome distributions in this species are zero heavy and censored (limited by the sperm storage time). Therefore they were analyzed by non-parametric tests, as recommended by Quinn and McKeough [36]. While this risks generating type II errors because of lower power, in most cases in the experiments reported below the non-significant results have high *p* values, with few between 0.05 and 0.2.

## Experiment 1: At Which Stage Does Fertilization Fail?

### Methods

To determine whether fertilization failure occurs before or after sperm-egg contact, the incidence of successes and failures judged from the presence/absence of embryos was compared with the incidence based on evidence for sperm-egg contact. Each female was placed in a wire mesh cage (38 cm wide X 23.5 cm deep X 18 cm height) with a male, using a different male for each female and allowing one completed mating with confirmed insemination. For half the females, the eggs laid on days 2–4 (the days of greatest fertilization probability) were collected, incubated for one week and broken open to check for embryos. For the other half of the females, the eggs laid on days 2–4 were collected shortly after laying (maximum delay of a few hours) and examined for sperm penetration holes in the inner perivitelline membrane, using published methods [37, 38]. (See reference [37] for the appearance of the holes in photomicrographs.) The yolk and albumen were separated and the yolk was placed in a dish and covered with PBS. A circle of perivitelline membrane about 1.5 cm in diameter with the germinal disk in the center was cut out with fine scissors, lifted off with two pairs of flat-tipped tweezers, washed with PBS, placed flat on a slide and examined immediately under dark-field microscopy at 4X for sperm penetration holes. Polyspermy is the norm in birds and multiple sperm penetration holes are expected if sperm-egg contact has occurred.

Two weeks later, the trials were repeated, using the same male-female pairings, but now with the egg examination method reversed, so that females whose eggs had been incubated following their first trial now had their eggs examined for sperm holes and vice versa. This design allowed a comparison of outcomes from the same set of females mated to the same males to see whether the percentages of failures judged from sperm holes and from embryos were similar holding bird identities constant. Because some males failed to achieve insemination, the mating trials were continued with new birds until there were 20 females (the *a priori* target *N*) that had been inseminated by the same male in both halves of the experiment.

### Results

**Fig 1:**
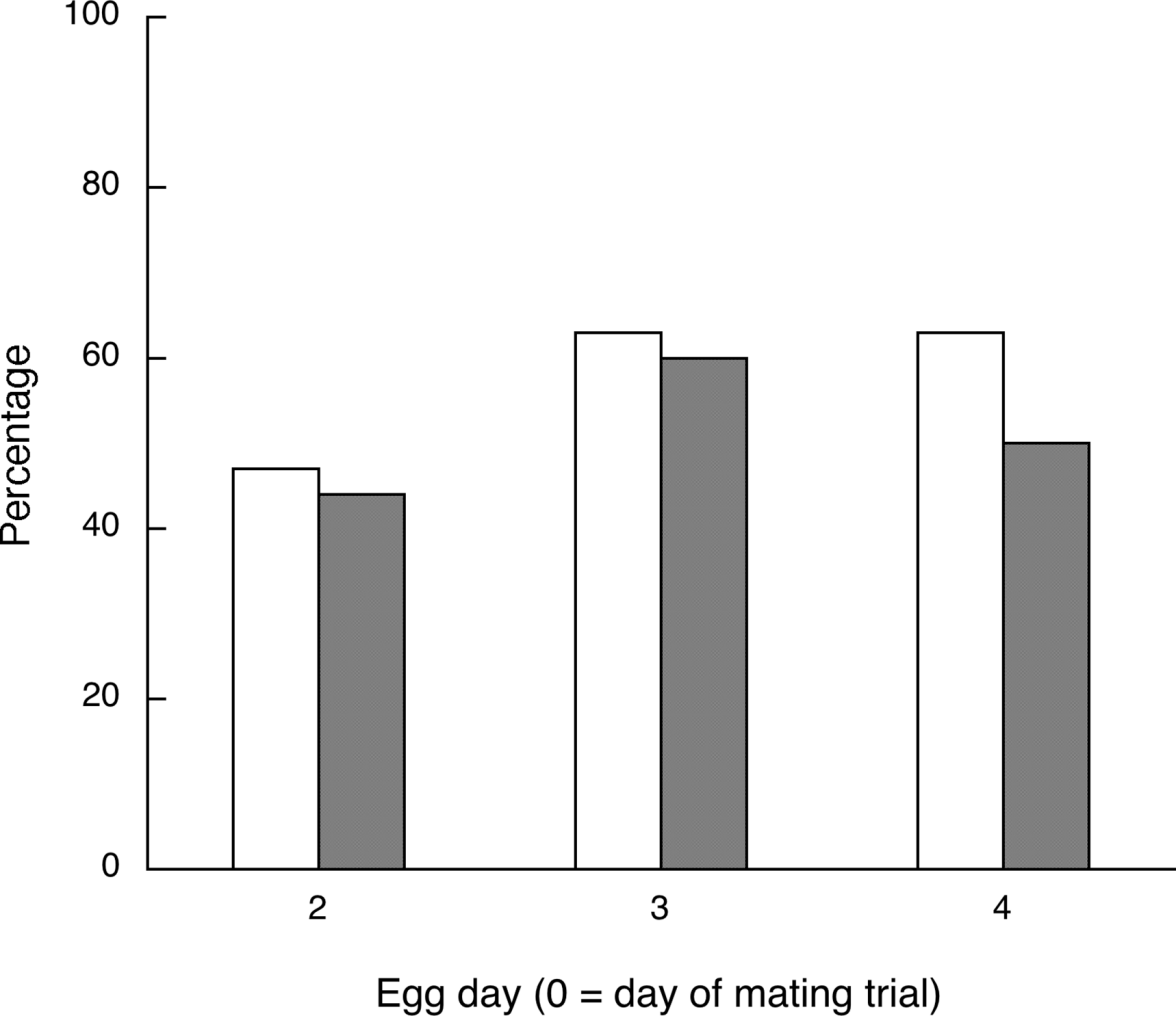
Experiment 1. Percentages of eggs laid by 20 females following a single insemination on days 2–4 of the laying sequence (day 0 = day of mating) that had sperm penetration holes in the perivitelline membrane (clear bars) or contained embryos after one week of incubation (gray bars). The fertilization failure percentages (100% minus the values shown by the bars) were 53% (day 2), 37% (day 3) and 37% (day 4) for sperm holes, and 56% (day 2), 40% (day 3) and 50% (day 4) for embryos. The fertilization outcomes were very similar for the two outcome measures.

The number of sperm holes in each egg ranged from 0 to 70, with a mean of 15 holes for the 34 eggs that had at least one hole. The percentages of eggs that had either sperm holes in the perivitelline membrane or an embryo following incubation for the three egg collection days are shown in Fig 1 (S1 Dataset). The percentages of successes and failures for each of the two outcomes were very similar and did not differ statistically for any of the three days (all McNemar’s binomial *p* > 0.6).

## Experiment 2: Fertilization Probability in Relation to a Prior Laying or Fertilization Gap

### Methods

Each female was placed in a wire mesh cage (38 cm wide X 23.5 cm deep X 18 cm height) with a male to allow one completed mating followed by confirmation of insemination. The population of birds used came from six different hatching cohorts, one cohort per year over six years. Trials were conducted until there were 185 trials with confirmed insemination, each with a different female and a different male.

Three versions of the overall hypothesis and their predictions were tested, as follows.

Hypothesis 1: sperm release is triggered by ovulation. Ovulation occurs shortly after oviposition. If no egg is laid, there was no ovulation the day before. Without ovulation, fewer sperm should be released. Therefore more should remain in the sperm storage tubules to be released for the next ovulation. It is predicted that either the next ovulation (i. e., the next egg laid following a day without an egg) should have a greater probability of being fertilized, or the last day on which a fertilized egg is laid should occur later (i. e., a longer sperm storage interval). In the latter case, there should be a negative relationship between the number of eggs laid and the last fertilized egg day.

**Fig 2:**
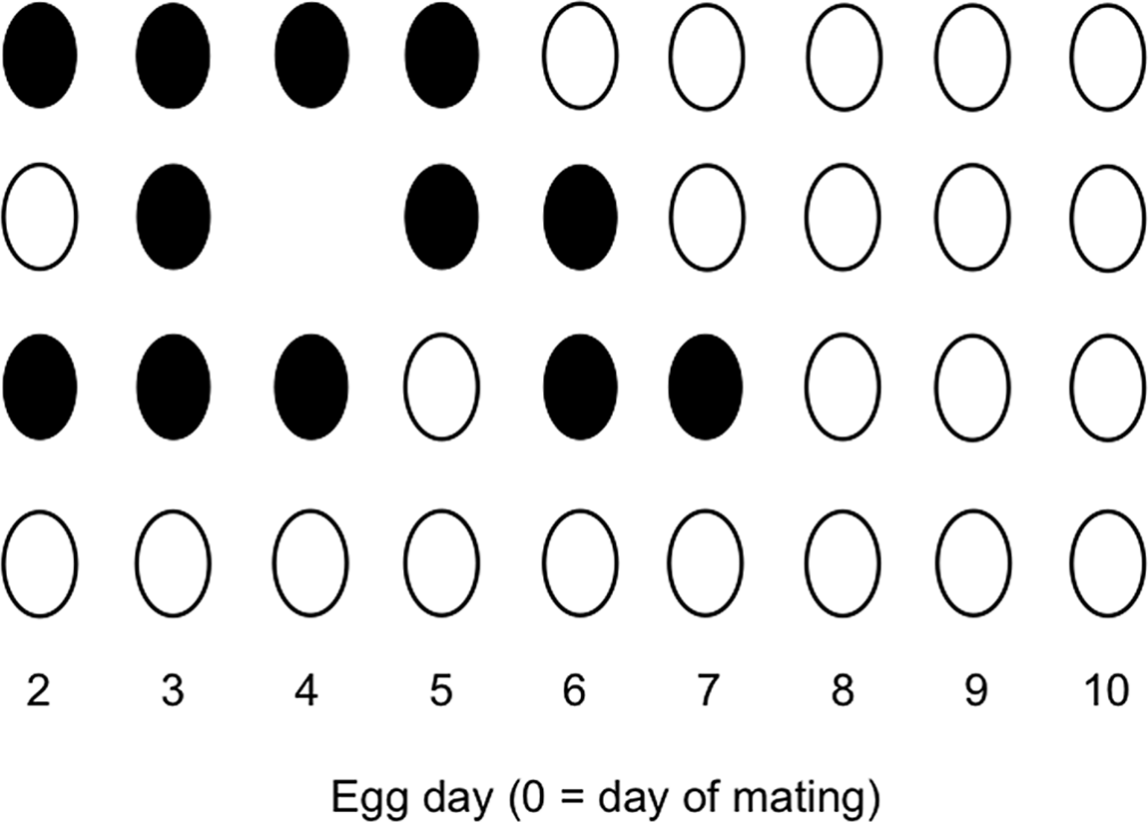
Experiment 2. Representative fertilization patterns in the eggs laid by four females (one female per row of eggs) on days 2–10 of the laying sequence following a mating and single insemination on day 0. Black ovals are fertilized eggs (those containing an embryo); white ovals are unfertilized eggs. The day 1 egg is never fertilized (it was ovulated before mating occurred) and day 11 is almost never fertilized (see Fig 3). The second row has a fertilization gap on day 2 and a laying gap on day 4. The third row has a fertilization gap on day 5. The fourth row (no fertilized eggs) was the outcome in 44% of the trials.

Hypothesis 2: sperm release is triggered by oviposition and fertilizes the ovum ovulated shortly after oviposition. If there is no egg laid, sperm will not be released and the next ovum will not get fertilized. The prediction is that the next egg following a day without an egg (a laying gap- see Fig 2) will have a lower probability of being fertilized. Alternatively, as with Hypothesis 1 the last fertilized egg day might occur later.

Hypothesis 3: for reasons unrelated to ovulation or oviposition, fertilization failure can occur if not enough sperm are released from the sperm storage tubules on a given day. If there is a fertilization gap (an unfertilized egg preceded and followed by a fertilized egg- see Fig 2), more sperm remain in the tubules for future ova, and so the last fertilized egg day should occur later.

Hypothesis 4: sperm continuously and randomly leak out of the sperm storage tubules in no particular relation to ovulation, oviposition or a prior fertilization gap. This is the passive sperm loss model proposed for chickens by [39]. The prediction is that fertilization probability will be unaffected by either a laying gap or a fertilization gap.

These hypotheses were tested by examining the egg laying and fertilization patterns of all the trials that produced at least one fertilized egg. To test Hypotheses 1 and 2, fertilization probabilities were compared for eggs laid on the same day relative to the mating day that did vs. did not follow a gap in laying (i. e., no egg laid the previous day). This comparison was made for each day except for day 2 (the day egg collection began) and days 10 and11 (when fertilization probability has dropped to near 0), using Fisher’s exact tests. To test the predictions from Hypotheses 1, 2 and 3 of a later day for the last fertilized egg, the last fertilized egg day was compared for trials with vs. without a laying or fertilization gap using t-tests for unequal variances. For this comparison, the few trials where the precise day of the last fertilized egg could not be known (because no eggs were laid later in the sequence) were not included.

### Results

**Fig 3:**
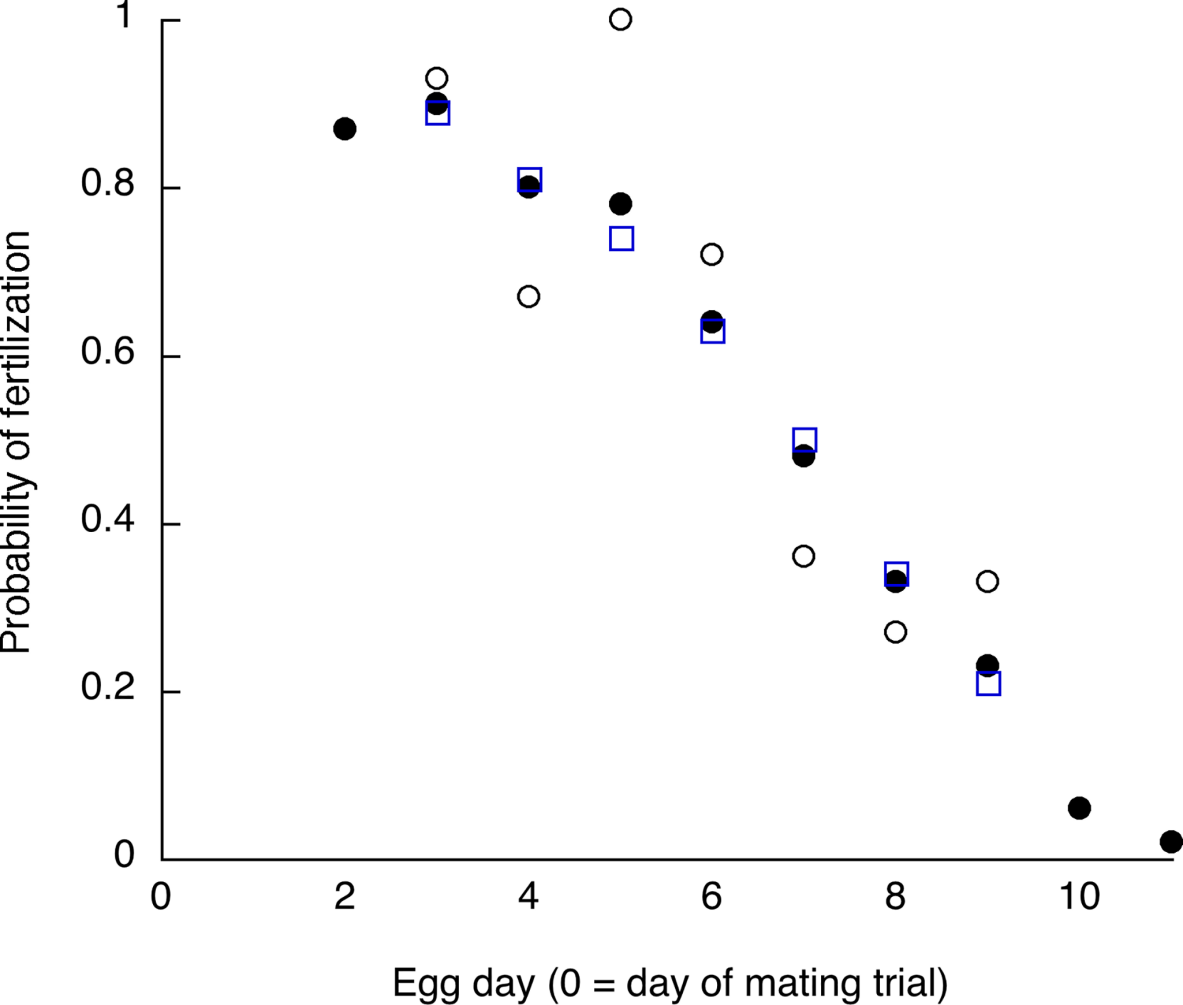
Experiment 2. Probability of egg fertilization on days 2–11 of the laying sequence for 103 singly inseminated females producing at least one fertilized egg. Solid circles: all eggs; open circles: eggs following a day when no egg was laid (a laying gap); open squares: eggs following a day when an egg was laid (no laying gap). There were no significant differences between those two probabilities on any of the days.

Of the 185 trials with confirmed insemination, 103 or 56% produced at least one fertilized egg; in the other 44% mating with insemination failed to fertilize any egg. For those 103 trials with at least one fertilized egg, the numbers of fertilized eggs ranged from 1 to 10 (11 to 100% of the eggs collected on days 2–11), with a mean of 4.5 (52%) and a median of 5 (50%) (S2 Dataset). Fig 2 shows four representative patterns of egg fertilization. Fig 3 shows the fertilization probabilities for each day in the sequence for the 103 trials with at least one fertilized egg. The probability of fertilization began to drop sharply after day 5 and was quite low (below 0.1) by day 10.

For the 103 trials with at least one fertilized egg, on any given day relative to the mating day there were six to 18 females that failed to lay an egg (mean = 14), creating a laying gap. Fig 3 shows fertilization probabilities for days 3 through 9 for those eggs that did vs. did not follow a laying gap. Those probabilities overall were nearly identical: 52/86 eggs fertilized or 0.605 fertilization probability if no egg had been laid the day before vs. 325/543 or 0.599 fertilization probability if an egg had been laid the day before. On Day 5 there was a non-significant trend (*p* = 0.06) for the fertilization probability to be greater following a laying gap; there were no statistically significant differences or trends on any other days (all *p* > 0.3) and across days probabilities following a laying gap were neither consistently higher nor lower (Fig 3). Nor was there any relationship between the last fertilized egg day and the number of eggs laid (Spearman’s rho = 0.0095, *p* = 0.93).

**Fig 4:**
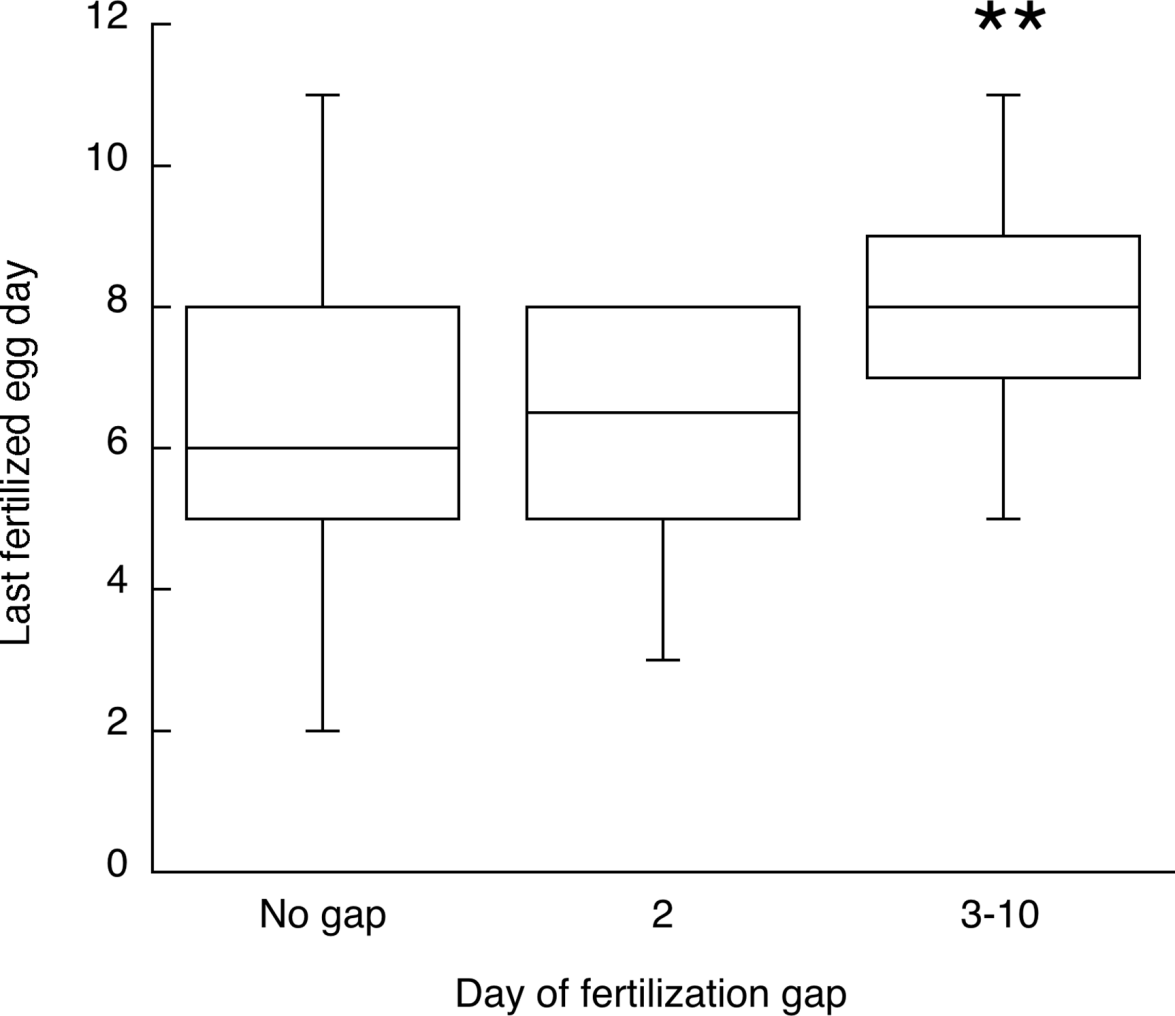
Experiment 2. The day in the laying sequence when the last fertilized egg was laid for trials/females with or without a fertilization gap on day 2 or later in the sequence. The box plots show the median, lower and upper quartiles and range. *N* = 37 trials for no fertilization gap, 10 for an unfertilized egg on day 2 and 35 for an unfertilized egg between days 3–10. ** *p* < 0.005 compared with trials with no fertilization gap.

A fertilization gap after fertilization had begun occurred in 44 (43%) of the trials. In another 11 trials, the first fertilized egg occurred on day 3 or later instead of day 2, creating another kind of fertilization gap. Three of those contained a fertilization gap later in the sequence as well. One female laid her only fertilized egg on day 6. Taken together, 50% of the trials produced some kind of fertilization gap. Fig 4 shows the last fertilized egg days for trials with and without a fertilization gap. The results are shown separately for cases where the gap occurred at the beginning of the sequence (i. e., a delayed start to fertilization, as in the second row of Fig 2) and those where fertilization began but a gap appeared later in the sequence (as in the third row of Fig 2). There was no statistically significant difference in the last fertilized egg day between trials with a day 2 gap and trials with no gap (Mann-Whitney *U* = 181, *p* = 0.93), but the last egg day was significantly later for trials with a gap after day 2 than for trials with no gap (*U* = 367, *p* = 0.0016).

## Experiment 3: Female Fertilization Gains from a Second Mating the Next Day

### Methods

This experiment compared fertilization outcomes for three groups of females in order to see if a second mating with insemination increased female reproductive success and, if so, by how much. Females in the Same Male group (*N* = 18) mated with a male on day 0 and mated again with the same male the next day (day 1). Females in the Different Male group (*N* = 18) mated with one male on day 0 and mated with a different male the next day (day 1). Females in the Control group (*N* = 18) mated with a male on either day 0 or day 1, with half assigned to each of the two days. Different males were assigned to the three groups of females. Mating trials were a day apart rather than spaced closely together because females are less receptive following mating, which would reduce insemination probability for the second mating. For a similar reason, all mating trials took place in the females’ home cages (wire mesh 51 cm deep X 25 cm wide X 25 cm height), a familiar environment, to increase the probability of receptivity and insemination. Eggs were collected on days 2 through 12, the last possible fertilized egg day.

As a follow-up to this experiment the Same Male and Different Male groups were repeated with new females (*N* = 20) and males but using a within-female design. Half the females were assigned randomly to each group for mating on two consecutive days and then two weeks later (after the sperm storage period had ended) group assignments were reversed and females were again mated on two consecutive days.

### Results

Four females did not have confirmed inseminations in one or both mating trials (two in the Same Male group and one in each of the other two groups) and were dropped from the experiment. Only one egg contained an early embryonic death, which was not counted as a successfully fertilized egg.

**Fig 5:**
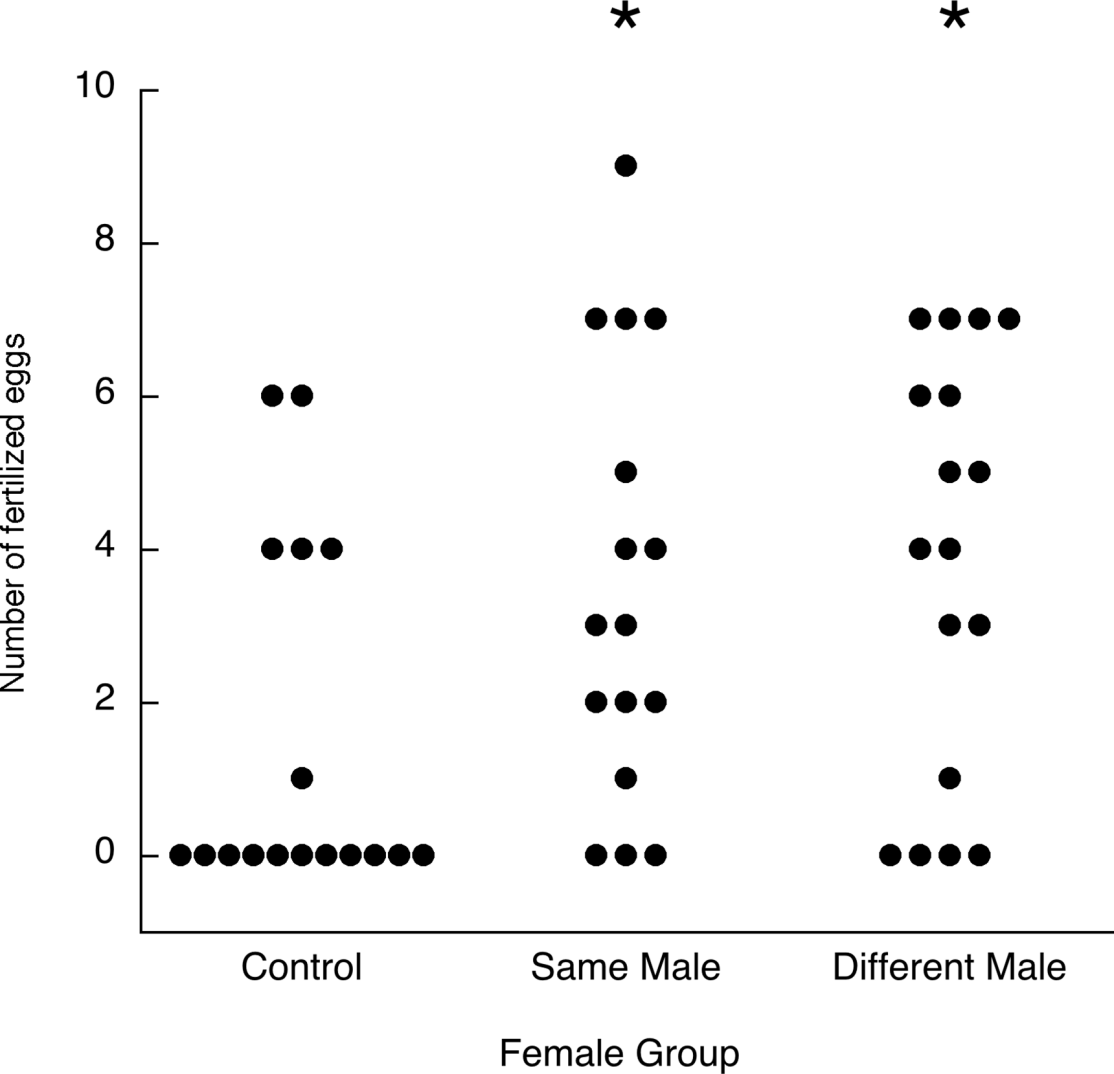
Experiment 3. Number of fertilized eggs laid by females inseminated by the same male on each of two consecutive days, a different male on each of two consecutive days, or one male on one day (control). Each dot is one female’s egg fertilization outcome. * *p* < 0.05 compared with the Control group.

Fig 5 shows the fertilization outcomes for the three groups. Even with two inseminations on consecutive days, some females did not lay any fertilized eggs (19% in the Same Male group and 24% in the Different Male group did not) and there were also many unfertilized eggs in the sequences with at least one fertilized egg. Nonetheless, both groups produced more fertilized eggs than the Control group (Same Male vs. Control: Mann-Whitney *U* = 74, *p* = 0.026; Different Male vs. Control: *U* = 75, *p* = 0.016) (Fig 5). The gain from two inseminations rather than one was mainly in the probability that there would be at least one fertilized egg, rather than in the number of eggs fertilized if any were fertilized. The proportions of zeros were significantly different in both two-mating groups compared to the control group (Same Male vs. Control: Fisher’s exact test *p* = 0.013; Different Male vs. Control: *p* = 0.037). The median numbers of fertilized eggs in sequences with at least one fertilized egg, on the other hand, did not differ significantly between two-mating and one-mating groups (medians 4, 5 and 4 for Same Male, Different Male and Control groups, respectively; both Mann-Whitney *U* > 0.3) (see Fig 5).

There was no statistically significant difference in outcomes between the Same Male and Different Male groups (*U* = 126, *p* = 0.72). In the within-female design follow-up experiment, 16 females had confirmed inseminations on all four days. The median numbers of fertilized eggs were 3.5 for the Same Male mating assignment and 3.0 for the Different Male assignment. Nine of the females laid more fertilized eggs in their Same Male assignment, five laid more in their Different Male assignment, and two laid the same number in each assignment. These outcomes were not statistically significantly different (Wilcoxon *T* = 41.5, *N* = 14, *p* = 0.50), nor did the proportions of zeros differ significantly in the two assignments (Same Male: 19%; Different Male: 31%; McNemar’s binomial *p* = 0.69).

## Experiment 4: Male Fertilization Gains from a Second Mating the Next Day

### Methods

This experiment compared fertilization outcomes of males mating with one vs. two females in order to see how much a second mating with confirmed insemination increased male reproductive success. Males in the Two Females group (*N* = 25) mated with one female on day 0 and a different female on day 1. Matings were a day apart to allow males to recover from any sperm depletion due to the first mating and to be comparable with female Experiment 3. Males in the Control group (*N* = 25) mated with one female, half on day 0 and half on day 1. Different females were used for each male. All mating trials took place in the females’ home cages as in Experiment 3.

### Results

**Fig 6:**
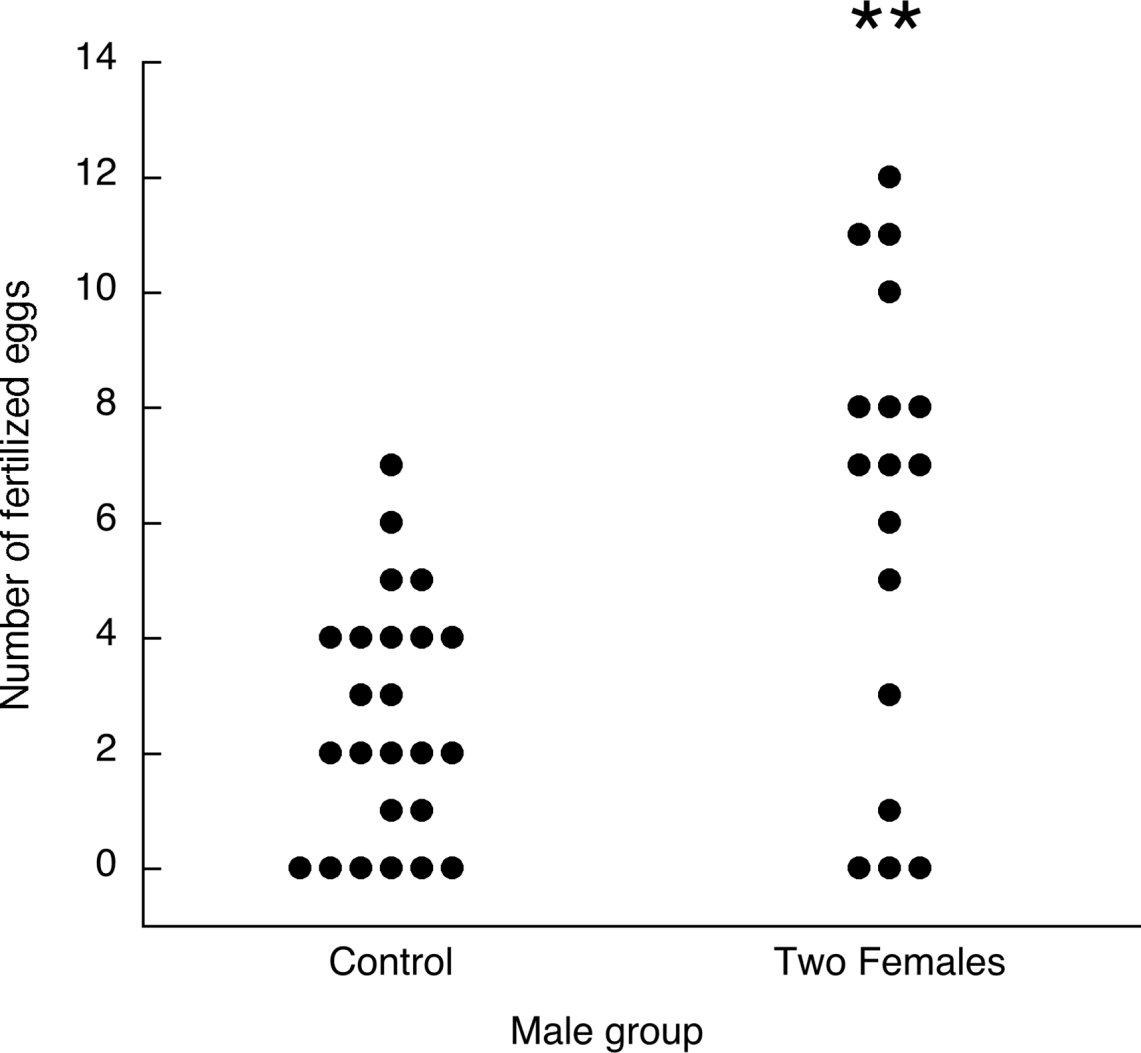
Experiment 4. Fertilization outcomes for males inseminating either two females, one on each of two consecutive days, or one female on one day (control). Each dot is the egg fertilization outcome for one male subject. ** *p* < 0.005 compared with Control group.

Eight males in the Two Females group and one male in the Control group failed to inseminate one or both of their assigned females, leaving 17 in the Two Females group and 24 in the Control group. There were eight eggs with an early embryonic death out of over 300 eggs total, which were not counted as successfully fertilized eggs. Fig 6 shows the fertilization outcomes for the two groups of males. While the proportions of zeros were not significantly different (3/17 and 6/24 for the Two Females and Control groups, respectively, *p* = 0.71), the median number of fertilized eggs was greater for the Two Females group than the Control group (medians including zeros: 7 and 2, respectively, *U* = 96, *p* = 0.0044; medians excluding zeros: 7.5 and 3.5, respectively, *U* = 33, *p* = 0.0004).

For the males in the Two Females group, there was no significant difference in fertilization outcomes between the first and second females (*T* = -39, *N* = 13, *p* = 0.68), confirming the absence of any order effect that could reflect incomplete sperm recovery or other carry over factors. Nor were the outcomes with the two females correlated (Spearman’s rho = 0.114, *N* = 17, *p* = 0.33).

**Fig 7:**
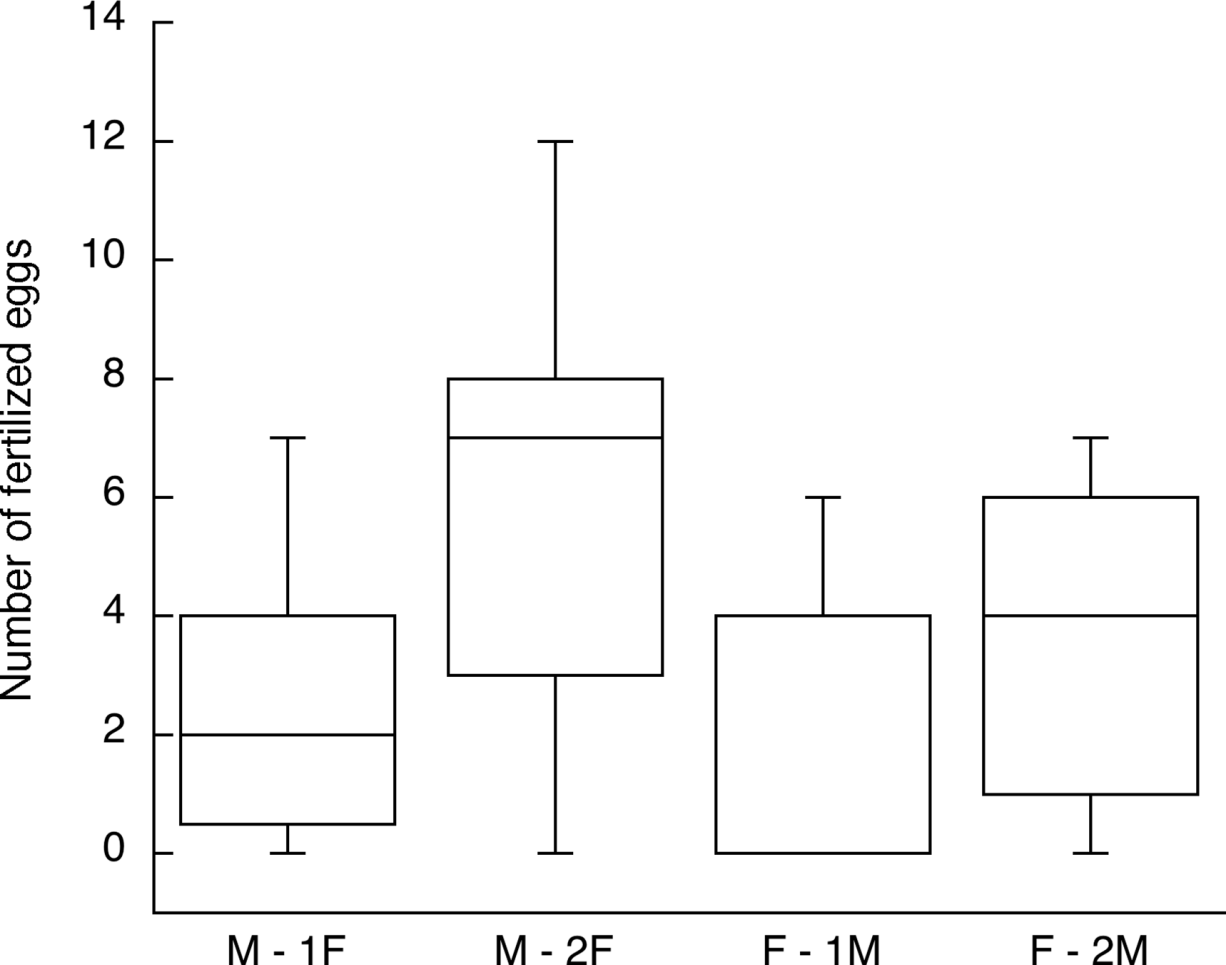
Fertilization outcomes for Experiments 3 (female subjects, F) and 4 (male subjects, M) summarized to show the sex comparison in reproductive success gains from a second mating with insemination. The M– 2F and F– 2M birds mated with a second opposite-sex bird on the next day; the M– 1F and F– 1M birds are the controls in the experiments that mated once on one day. The box plots show the median, lower and upper quartiles and range. Both sexes gained from the second mating on average, but some subjects still had zero success (see text and Figs 5 and 6).

Fig 7 summarizes the fertilization outcomes for Experiments 3 and 4 so that the gains from a second mating can be compared between male and female subjects mating with a different partner on the second day. The median numbers fertilized increased from 2 to 7 for males and 0 to 4 for females. Because of the shapes of the distributions, it is not possible to compare these gains quantitatively, nor does a change in median from 2 to 7 mean that males gained more than three times the success. Nonetheless, it is clear that on average both sexes gained substantial additional reproductive success while still failing to produce a single fertilized egg in some cases.

## Experiment 5: Do Males Gain Any Fertilizations from Having Two Females Present Simultaneously?

### Methods

Here the fertilization outcomes of males were compared when they had two females to mate with at the same time vs. one female, using a within-male design. Each of 60 males had two mating trials, one on each of two consecutive days. On day 0, half the males had two females to mate with (Two Females trials) and half had one female (One Female trials). On day 1 the trial type assignments were reversed, so that trial type order was counterbalanced. Different females were assigned to each male. Trials were conducted in an otherwise empty room in a large wire mesh cage 1.8 m X 0.8 m X 0.9 m (height) with three small wire mesh release cages inside, two in the rear corners and one in the front center. For each trial one female (One Female trials) or two females (Two Females trials) were placed in the rear release cages, alternating left and right cages for One Female trials. Then the male was placed in the front release cage. After 30 sec the male was released by pulling on a rope attached to his release cage from behind a blind and allowed to move freely in the testing arena. After 30 sec the female(s) were released and all birds observed from behind the blind for 5 min, noting whether the male attempted to mate with one or both females and in what order he completed a mating. Attempted to mate was defined as grabbing the feathers of the head or neck of the female, the first act in the male quail copulatory sequence. Completed a mating was defined as sudden cessation of the mating attempt following a cloacal contact.

### Results

Twelve males showed no interest in mating in one of their trials and were dropped from the experiment, leaving 48 males as subjects that attempted to mate in both of their trials (S3 Dataset). In the Two Female trials, 33 males (69%) attempted to mate with both females. The percentages of trials that resulted in confirmed insemination (foam positive females) were as follows: One Female trials, 71%; Two Females trials, 81% with one foam positive female and 27% with two foam positive females. Ten males inseminated all three females across their two trial types. For those 10 males the median numbers of eggs fertilized were the same (median 3) in the two trial types, with no statistically significant difference (Wilcoxon *T* = 12, *N* = 8, *p* = 0.46). Twenty-eight males inseminated one female in their Two Females trials in addition to the female in their One Female trial. For those males the median numbers of eggs fertilized were also the same (median 3) in the two trial types, with no statistically significant difference (*T* = -101, *N* = 20, *p* = 0.898).

Thirteen males inseminated both females in their Two Females trials. The numbers of eggs fertilized from the first and second of the two matings tended to differ (medians 3 and 0, respectively, *T* = -7.5, *N* = 9, *p* = 0.074) but a more pronounced result was that the outcomes with the two females were positively correlated (Spearman’s rho = 0.48, *p* = 0.048). Finally, if all trials for the 48 males are included, regardless of whether there was a confirmed insemination, and foam negative trials are counted as zero eggs fertilized, males did not fertilize significantly more eggs in their Two Females trials (medians 1 and 0.5 for Two Females and One Female trials, respectively; *T* = 199.5, *N* = 29, *p* = 0.70). Nor was there any significant difference in the percentages of trials that did or did not fertilize any egg; at least one egg was fertilized in 52% of the males’ Two Female trials and 50% of their One Female trials (McNemar’s binomial *p* = 1.0). Having two females to mate with in the same trial neither increased nor decreased the males’ fertilized egg numbers. Although a small number of males fertilized many eggs that way (a total across the two females of up to 13 eggs), others failed to fertilize a single egg in either female in spite of inseminating both.

## Discussion

These experiments provide new information about some of the events and mechanisms of the relationship between insemination and fertilization that are relevant to understanding post-copulatory sexual selection. The relationship has a strong probabilistic element (“hit or miss”) and even mating twice still leaves many eggs unfertilized. The generation of a large data set has allowed several hypotheses about the nature of sperm release from storage to be tested, with support for one (Hypothesis 3 of Experiment 2). Males and females are shown to gain to a similar extent by a second mating the next day, but males do not realize any gain by having two females at the same time to mate with.

In Experiment 1 the fertilization outcomes determined by the presence of embryos at one week of incubation were very similar to the outcomes determined by the presence of sperm penetration holes in perivitelline membranes of freshly laid eggs. In addition, the incidence of early embryonic deaths was quite low in all the experiments. Thus most of the failures of inseminations to result in reproductive success, a pronounced phenomenon in this species, are due to failures of any sperm to reach and penetrate ova, not to failures occurring after that stage. Either an insufficient number of sperm were stored, stored sperm were not released in sufficient numbers, or an insufficient number of released sperm reached the site of fertilization in the oviduct. In artificial insemination of chickens, the number of sperm inseminated predicts the number stored [40]. What then determines the number inseminated during a natural mating? One possibility is the duration of the cloacal contact, which would depend both on the male’s copulatory ability and on how long the female holds still during copulation [41]. A longer duration might allow for more sperm to be transferred and/or for the female to regulate placement of the ejaculate in her reproductive tract. In quail female receptivity is not essential for cloacal contact to occur. Although males have more difficulty inseminating unreceptive females, if they do achieve insemination they fertilize similar numbers of eggs [21, 42].

In Experiment 2 the probability that an egg would be fertilized was not related to whether the female had laid an egg the previous day. For some time there have been mixed opinions about whether sperm release from the SSTs is related to the events of the daily ovulation/oviposition cycle in birds [43, 44]. Bakst [30] thought egg rotation in the lower oviduct might affect sperm storage or release, and Ito et al. [45] found that an injection of progesterone, a hormone that peaks pre-ovulation, stimulated sperm release from SSTs in chickens. Grigg [32] and Bobr et al. [31] thought passage of the yolk might cause release of sperm from the infundibular sperm storage area, but Stepinska and Bakst [44] doubted that there is any true sperm storage in the infundibulum. Burke and Ogasawara [39] proposed that sperm are released slowly, continuously and passively from the SSTs. Data from chickens [46] and from female quail removed from mixed-sex housing [16] seem to support that hypothesis.

In Experiment 2, the fertilization probabilities over time, derived from single inseminations, are very similar to those reported by Birkhead and Fletcher [16] for female quail that had presumably been mating daily several times per day. In both sets of results the fertilization probability drops off sharply after day 5, becomes quite low (less than 0.2) by day 10 and reaches 0 at day 11. The similarity in the sperm storage interval (duration of fertilization in the egg sequence) in spite of the difference in the number and timing of inseminations is consistent with the conclusion of Birkhead and Fletcher [16] that the sperm storage interval is mainly due to the rate of loss of sperm from SSTs rather than to the number of sperm stored. Also consistent with this conclusion are the results of Experiment 3, in which two inseminations increased the likelihood that the female would lay at least one fertilized egg (a function of the number inseminated and stored) but not the number of fertilized eggs laid if at least one was laid (reflecting the sperm storage interval, a function of sperm loss rate).

The results of Experiment 2 with respect to fertilization gaps, however, suggest that sperm release from the SSTs is not always continuous. The last day on which a fertilized egg was laid occurred later if there had been an unfertilized egg between two fertilized eggs, as if sperm release/loss had been interrupted temporarily by some unknown factor. Also, female mammals do not store sperm, yet as in Experiment 3 mating multiple times increases the probability of a pregnancy (in quail, the probability of at least one fertilized egg) more than it increases litter size (number of fertilized eggs) [47]. In both birds and mammals, it is as if a threshold number of sperm are required for any fertilization to occur regardless of whether there is sperm storage.

In Experiments 3 and 4 both sexes on average gained fertilization success from a second insemination the next day. Yet even with two inseminations there were still a number of complete fertilization failures (24% in the female experiment and 18% in the male experiment). When the males were the subjects, the outcomes with each of the two females were uncorrelated; it was “hit or miss” each day, with day 2 providing a second independent chance to “hit.”

The results of Experiments 3 and 4 can also be viewed in the context of Bateman’s third principle that males gain more by mating multiply than females do [48, 49]. The similar male and female average gains from mating twice (Fig 7) suggest that Japanese quail might not fit that principle. Indeed, there is increasing evidence that the principle does not apply universally and instead that the size of the sex difference, if any, is quite variable across species. In avian research, there have been reports of gains by females or very similar gains by both sexes (e. g. [50, 51]), including some results from other galliform birds (e. g. [52]). At the same time, work with yet other species supports the sex difference of Bateman’s original principle (e. g. [53]. Collett et al. [54] explained that some such reports might be misleading because, for example, they do not include matings that did not result in fertilization or are biased by male preferences for more fecund females. Here the quail experiments were designed so that all females were good egg producers, mate choice was minimized and the exact number of matings was known. Whether either sex of wild quail is under selection to mate with multiple partners is unknown, however. The females in Experiment 3 benefitted as much from mating with the same male twice as from mating with two males, making fertilization insurance an equally plausible explanation for why females might mate multiple times [55–57]. In a study of wild female collared flycatchers (*Ficedula albicollis*), repeated inseminations were also required for an entire clutch to be fertilized [58]. If there is any advantage to female quail of mating with multiple males, as opposed to multiple matings with the same male, it is more likely to occur at later stages of reproductive success, such as hatching success and offspring survival.

Males were not able to gain fertilizations by having two females to mate with in the same trial (Experiment 5). The second female neither helped nor hurt their fertilization success. Male Japanese quail are enthusiastic copulators and many subjects were highly motivated to try to mate with both females, often running back and forth between them. Few males (only 27%) were able to inseminate both females, however, and even those that were able to did not fertilize more eggs in total than they did in their trials with a single female. In spite of their high sperm production rate [25], male quail are limited in the ability to realize greater reproductive success with multiple female partners over such a short time scale. Male zebra finches were shown to have a similar sperm limitation when presented with two model females simultaneously [59]. While that is perhaps less surprising in a species that is quite genetically monogamous in the wild [60], it also suggests that sperm limitation may be fairly general in birds independently of mating system.

Can females gain fertilized eggs by mating with two males in quick succession? The act of copulation tends to reduce female receptivity [18] but because males engage in forced copulations females can sometimes be inseminated by two males close together in time. In a recent study of the role of male quail foam in sperm competition, using similar mating procedures and the same strain of quail, females were mated with two males only 4 min apart [19]. In 63% of the 132 trials, both males completed mating, as judged from their behavior, and in 83% of those double mating trials, there was at least one fertilized egg. That is a much higher percentage than for the females in Experiments 2 and 3 that were mated once (56% and 35%, respectively), a sizable difference that is unlikely to be entirely due to the minor procedural differences between the mating trials in the studies. Such a gain is consistent with the conclusion reached above (in the discussion of Experiment 3) that whether any eggs are fertilized is a function of the number of sperm inseminated [5, 16, 61]. Even with some interference due to the role of the foam in sperm competition, there would probably still be more sperm to store following two males mating close together in time than following one. Thus it seems that females can gain fertilizations much more than males can by mating with two partners in quick succession, whereas both gain to a more similar extent if the two matings are a day apart.

Overall, the experiments reported here provide new insights into the relationship between single and double natural inseminations and fertilization outcomes in a sperm-storing vertebrate uniquely well suited to such research. Although both sexes gain fertilization success by two inseminations a day apart, even double inseminations have a rather limited capacity to fertilize a significant number of eggs, so that multiple mating would be needed to ensure fertilization of an entire clutch. The few studies of wild *Coturnix* quail (both *Coturnix coturnix* and *C*. *japonica* with recent wild-caught ancestry) suggest a flexible mating system with opportunities for multiple mating [34, 35]. The results of the experiments also highlight a paradox in the reproductive success of male Japanese quail. The low fertilization success of inseminations co-occurs with several other male characteristics that suggest past sexual selection for increased fertilization success, including high and fairly indiscriminate sexual motivation [62], tendency to engage in forced copulations [21], the unique foam that aids sperm competition [19], large testes (e. g. [24] and atypical sperm morphology [26]. Studies of other *Coturnix* species, some of which are reported to be monogamous [28], are needed to see whether these characteristics tend to be associated across species and how they are related to variation in mating system.

## Supporting Information

S1 ARRIVE Checklist (PDF)

S1 Dataset Experiment 1. (XLSX)

S2 Dataset Experiment 2. (XLSX)

S3 Dataset Experiment 5. (XLSX)
